# A Hybrid Robust Image Watermarking Method Based on DWT-DCT and SIFT for Copyright Protection

**DOI:** 10.3390/jimaging7100218

**Published:** 2021-10-19

**Authors:** Mohamed Hamidi, Mohamed El Haziti, Hocine Cherifi, Mohammed El Hassouni

**Affiliations:** 1 LIB EA 7534, Faculté des Sciences Mirande, 9 Avenue Alain Savary, BP 47870, 21078 Dijon, France; hocine.cherifi@u-bourgogne.fr; 2 Higher School of Technology, Mohammed V University in Rabat, Rabat 1040, Morocco; elhazitim@gmail.com; 3 Faculté des Lettres et des Sciences Humaines, Mohammed V University in Rabat, Rabat 1040, Morocco; mohamed.elhassouni@flsh.um5.ac.ma

**Keywords:** robust image watermarking, scale-invariant feature transformation (SIFT), geometric distortions, DWT, DCT

## Abstract

In this paper, a robust hybrid watermarking method based on discrete wavelet transform (DWT), discrete cosine transform (DCT), and scale-invariant feature transformation (SIFT) is proposed. Indeed, it is of prime interest to develop robust feature-based image watermarking schemes to withstand both image processing attacks and geometric distortions while preserving good imperceptibility. To this end, a robust watermark is embedded in the DWT-DCT domain to withstand image processing manipulations, while SIFT is used to protect the watermark from geometric attacks. First, the watermark is embedded in the middle band of the discrete cosine transform (DCT) coefficients of the HL1 band of the discrete wavelet transform (DWT). Then, the SIFT feature points are registered to be used in the extraction process to correct the geometric transformations. Extensive experiments have been conducted to assess the effectiveness of the proposed scheme. The results demonstrate its high robustness against standard image processing attacks and geometric manipulations while preserving a high imperceptibility. Furthermore, it compares favorably with alternative methods.

## 1. Introduction

The growth of digital information technologies makes their distribution and duplication much easier. Therefore, the necessity to design secure techniques has increased in the last few decades. Digital image watermarking has been found to be an effective solution for copyright protection of images [1]. Its basic procedure is to embed imperceptible information, termed watermark, in the original image. Thus, the copyright of the image can be provided by extracting the embedded secret watermark.

Three main properties are required in image watermarking systems [2]: imperceptibility, capacity, and robustness. Imperceptibility refers to the fact that the watermarked image should look identical to the original one. Capacity represents the maximum number of bits embedded in the original image. It is the primary constraint that should be ensured after imperceptibility [3] in high-capacity methods. Indeed, in this category of techniques, a considerable quantity of information should be embedded without losing image quality. In the copyright protection methods, this constraint is less critical, but it can influence the results in terms of robustness of imperceptibility, especially when the watermark size is too big. Robustness refers to the ability to detect the watermark even if the watermarked image suffered from several manipulations called attacks. A good watermarking scheme should ensure the best trade-off between these three properties. Indeed, generally, with the increase of capacity, the robustness of the image decreases while simultaneously decreasing its imperceptibility and vice versa. The main objective of the proposed method is copyright protection. Therefore, capacity is not a primal issue. Indeed, the main requirements for copyright protection applications are imperceptibility and robustness.

Watermarking schemes can be categorized according to embedding domain, extracting technique, watermark robustness. The embedding domain can be spatial or a transform domain. Spatial methods [4] embed the watermark by directly altering the pixels, while the transform domain methods embed the watermark after performing a transformation, such as DFT [5,6], DCT [7], DWT [8]. The extraction method can be a non-blind, or semi-blind, or a blind technique. The non-blind methods [9] require the original image in the extraction process. The semi-blind methods need the watermark and some side information. The blind techniques [10] need only the secret key to extract the watermark. In terms of robustness, the watermarking technique can be fragile [11], semi-fragile [12] or robust [13,14]. Fragile watermarking schemes are designed to be weak to attacks, including malicious tampering and common processing. They have been proposed specifically for integrity verification and image authentication [15]. Semi-fragile techniques are used with the aim of detecting any unauthorized modification while resisting some image-processing operations [16,17]. Robust watermarking methods designed for copyright protection should resist a wide variety of common attacks, especially malicious attacks, including filtering, noise, lossy compression, geometric attacks, etc.  [18,19]. In copyright protection applications, there is no need to share the side information (secret key, scale-invariant feature transformation (SIFT) descriptor, etc.) [20]. Indeed, the owner of the image is the only one who needs to possess this information. In case of dispute, he uses it to prove the ownership of the image [4,21].

In this paper, a SIFT-based robust watermarking method using DWT and DCT is proposed. The DWT is used due to its excellent spatial localization and multiresolution characteristics, which are similar to the human visual system (HVS) [22]. The reason behind using DCT is its strong energy compaction property [23] and good robustness against common image processing attacks. Thanks to the combination of these two techniques, the proposed method can withstand common signal processing attacks such as filtering, noise and JPEG compression, among others. Furthermore, the use of SIFT ensures robustness against geometric attacks. SIFT is used in the extraction stage to correct the geometric attacks, including rotation, translation, and scaling. The watermark is inserted in the DCT middle band of the HL first level DWT band of the original image. In fact, for each block 8×8, it consists of modifying 22 middle band DCT coefficients of the LH first level of DWT band. The extraction process is quite simple. It is sufficient to calculate the SIFT features to synchronize the attacked image to correct geometric attacks. The scheme is semi-blind since the SIFT descriptor is needed in the extraction process. Thanks to its resistance against RST attacks, the SIFT descriptor has been used in order to resist geometric attacks. Afterward, the watermark bits are extracted using the inverse processing of embedding. We note that a securing step can be incorporated in the proposed watermarking scheme using Arnold transform [24] or a more sophisticated cryptographic technique [25]. We note that the method is blind if no-geometric distortions are performed because no extra side information is necessary for the extraction phase. Only the secret key is needed to extract the watermark.

The proposed method has been compared to the state-of-the-art methods. The main criteria guiding the choice of alternative methods is to perform a fair comparison with similar methods presenting a comparable level of complexity. Hybrid methods combining several transformations [26,27,28], as well as SIFT based methods [13,29], are used for comparative evaluation, while [21] has been used since it has the same application as the proposed method (copyright protection).

The rest of this paper is as follows. Section 2 presents the related works. In Section 3, SIFT, DCT, and DWT are briefly explained. Section 4 describes the proposed watermarking method in details. Section 5 discusses the experimental results. Finally, the conclusion is given in Section 6.

## 2. Previous Work

The state-of-the-art image watermarking methods discussed in this paper can be classified into three categories: single transform-based method, hybrid transform-based methods, and scale-invariant methods.

In [21], the authors proposed a digital image watermarking method based on singular value decomposition (SVD). Firstly, the SVD is applied to the original image to obtain the orthogonal matrices U and V and the diagonal matrix S. Then, the watermark is embedded into the diagonal matrix S additively. The watermarked image is reconstructed using the modified matrices Sw Uw and Vw. Experimental results show that the method gives good results in both security and robustness against several attacks, such as compression, filtering, noise, cropping, etc.

In [30], an image watermarking method using contourlet transform along with singular value decomposition is proposed. In the embedding, the contourlet transform is applied to the original image, and the coefficients are modified by combining singular values of the selected direction with singular values of the watermark. The technique gives good imperceptibility results and is robust against several attacks. The method proposed by Amini et al. [31], presented a robust multiplicative watermark decoder using vector-based hidden Markov model in the wavelet domain is proposed. The results show good resistance to attacks and ensure a good imperceptibility. Based on single transformations, several transforms can be used as watermarking primitives. In [32], the authors proposed a color image watermarking scheme using quaternion polar harmonic transform (QPHT) with a maximum likelihood decoder. The watermark is embedded into the QPHT magnitudes using a multiplicative approach. The method ensures both imperceptibility and robustness. In [33], a color image watermarking scheme in the sparse domain is presented. The method considers the inter-channel dependencies between RGB channels and inter-scale dependencies of the sparse coefficients of color images by employing the hidden Markov model.

The main objective of the majority of existing watermarking schemes is to provide good robustness against several attacks preserving at the same time a high imperceptibility. Hybrid methods generally perform better than single transform methods. As a consequence, the need to develop these methods that combine two transforms to achieve this aim has increased considerably. Several hybrid methods have been proposed in the literature [34,35,36,37,38].

In Lagzian’s method [26], singular values of the redundant discrete wavelet transform (RDWT) are modified to insert the watermark. Makbol et al. [27] proposed a hybrid method based on integer wavelet transform (IWT) and singular value decomposition (SVD). The authors embed the watermark in the singular values of the first level of IWT.

Singh et al. [28] proposed a hybrid semi-blind method in the redundant wavelet domain. The authors take advantage of the shift-invariance of “RDWT” and nonsubsampled contourlet transform (NSCT) to avoid the shift sensitivity of the classical wavelet transforms. The watermark is inserted by modifying the SVD coefficients in the RDWT-NSCT domain.

Hybrid schemes are generally very robust against a wide range of attacks, especially image processing operations, since they exploit the benefits of two or more transformations to achieve watermarking robustness. Nevertheless, the majority of these methods show weakness to geometric attacks. To overcome this issue, methods using invariant descriptors like SIFT [39] and SURF [40] have been widely used. SIFT has been extensively proposed for image watermarking against geometric attacks [13,41,42,43]. In [41], a robust scheme against resolution scaling has been proposed. First, a watermark zone selection algorithm is performed to get the candidate pixel locations that are to be modified. Afterward, SIFT features, which act as a watermark, are extracted and registered. Then, a patch is embedded in the image such that it gives robust SIFT features.

In [42], the watermark is embedded into the circular patches invariant to scaling and translation, generated by the SIFT descriptor. The authors take advantage of the polar-mapped circular patches to ensure rotation invariance.

A rotation, scale, and translation invariant watermarking scheme based on discrete Tchebichef transform (DTT), singular value decomposition (SVD), and scale-invariant feature transform (SIFT) is proposed [44]. The DTT coefficients of the image are arranged similarly to the sub-band scheme generating LL, HL, LH, and HH sub-bands. The principal components of the watermark are inserted into the diagonal components of each DTT sub-band. Next, Arnold transform and permutation applied to the watermark are used to enhance security. The scheme is robust to geometrical and combined attacks.

Chen et al. [45] proposed a robust watermarking scheme with a feature-based synchronization technique. The watermark is repeatedly embedded in each selected local square feature region (LSFR) by modulating the discrete Fourier transform (DFT) coefficients. The extraction is based on a local statistical feature, and the SURF orientation descriptor is used for watermark synchronization. The method is robust against common attacks and screen-cam attacks. The method is effective against screen-cam, as well as common desynchronization attacks.

In [29], a robust watermarking scheme using (SIFT) and (DWT) domain is proposed. The SIFT feature areas are extracted from the original image, and  one level DWT is applied on the selected SIFT feature areas. Differently to the proposed approach, which embeds the watermark in a single sub-band, they insert the mark in the two sub-bands HL1 AND LH1. To do so, the watermark is divided into two parts that are inserted by modifying the fractional portion of the horizontal or vertical, high-frequency DWT coefficients. The experimental results showed that the scheme can resist both signal processing and geometric attacks.

The authors of [43] proposed robust image watermarking based on scale-invariant feature transform (SIFT), singular value decomposition (SVD), and all phase biorthogonal transform (APBT). A series of SIFT keypoints are obtained after carrying out SIFT, which are selected to obtain the neighborhood that can be used in the watermark embedding process. A block-based APBT is performed on the neighborhoods of the selected feature points. To insert the watermark, a coefficients matrix of a set of APBT coefficients for SVD is generated.

In [13], a SIFT-based watermarking scheme in the DWT-SVD domain is proposed. First, a 3-level discrete wavelet transform (DWT) is performed to the original image. Next, the SVD is applied to the LL${}_{3}$, and the watermark is embedded additively. The rotation, scale, and translation (RST) attacks are corrected by matching the key points of the original image and the watermarked one.

Recently, a SURF-DCT based image watermarking has been proposed [46]. First, the watermark is encrypted using chaotic encryption technology in order to enhance its security. Next, the DCT coefficients are modified using the positive and negative quantization rules. The method proves to be resistant against geometric and non-geometric attacks.

In our previous work [24], we proposed a blind robust image watermarking method based on the discrete Fourier transform (DFT) and DCT for copyright protection. The watermark is inserted in the DCT middle band of the DFT magnitude. The watermark is encrypted with the Arnold transform to increase the security of the proposed method. The method shows high imperceptibility for textured and non-textured images. Regarding the robustness, the technique can withstand signal processing attacks, JPEG, JPEG2000 compressions, etc., but shows weakness to geometric attacks. To overcome this problem, we propose a novel method based on SIFT to avoid vulnerability to these attacks.

## 3. Background

This section describes three techniques relevant to the proposed method, namely, the DCT, DWT, and SIFT. The DWT and DCT techniques are used to embed the watermark bits, while SIFT is used to make the proposed method invariant to geometric attacks.

### 3.1. Discrete Cosine Transform

The discrete cosine transform (DCT) is a famous transformation techniquee that transforms an image from the spatial domain to the frequency domain [47]. It has been widely applied in image processing exploiting both the decorrelation and the energy compaction properties. The mathematical expressions of the 2D-DCT and inverse 2D-DCT are, respectively:

(1) $$\begin{array}{c} C\left(u,v\right)=\frac{2}{\sqrt{mn}}\alpha \left(u\right)\alpha \left(v\right)\sum_{x=0}^{M-1}\sum_{y=0}^{N-1}f\left(x,y\right)\times \\ cos\frac{(2x+1)u\pi }{2m}\times cos\frac{(2y+1)v\pi }{2n} \end{array}$$

(2) $$\begin{array}{c} f\left(x,y\right)=\frac{2}{\sqrt{mn}}\sum_{u=0}^{M-1}\sum_{v=0}^{N-1}\alpha \left(u\right)\alpha \left(v\right)C\left(u,v\right)\times \\ cos\frac{(2x+1)u\pi }{2m}\times cos\frac{(2y+1)v\pi }{2n} \end{array}$$

where f(x,y) and C(u,v) are the pixel values in the spatial domain and the DCT coefficients, respectively. m,n represent the block size. α(u) and α(v) are two coefficients defined as follows:

(3) $$\alpha \left(u\right)\alpha \left(v\right)=\left\{\begin{array}{cc} \frac{1}{\sqrt{2}} & iff(u,v)=0\\ 1 & else \end{array}\right.$$

### 3.2. Discrete Wavelet Transform

**Figure 1:**
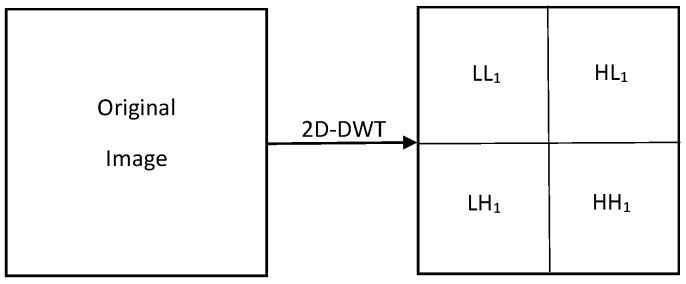
1-level decomposition of the 2D-DWT.

Discrete wavelet transform has been widely used in image processing and its applications. It consists of decomposing an image into four sub-bands, one corresponding to the low pass band (LL) and three others corresponding to horizontal (HL), vertical (LH), and diagonal (HH) high pass bands. The image can be decomposed iteratively by further decomposing the low pass band each time. It has been used extensively in image watermarking due to its excellent spatio-temporal localization as well as its correlation with the human visual system (HVS) [48]. Figure 1 depicts one-level decomposition of the 2D-DWT.

### 3.3. Scale Invariant Feature Transform (Sift)

**Figure 2:**
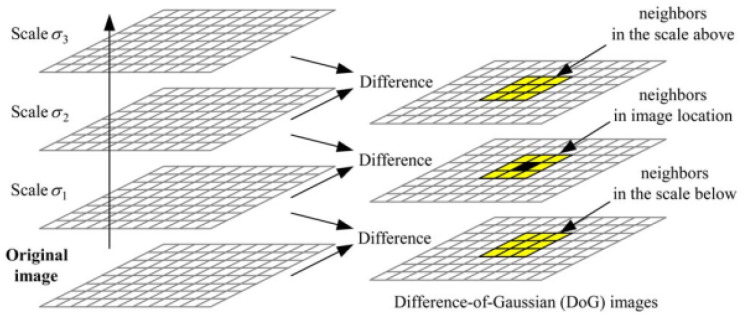
Difference-of-Gaussians (DoG) function and neighbors of a pixel [42].

The scale-invariant feature transform (SIFT) proposed by G. Lowe [39] is an image descriptor that extracts characteristic features. These features are invariant to image translation, rotation, scaling, and brightness change. Firstly, a search for peaks in the scale space of the difference-of-Gaussians (DoG) function is performed to select the candidate’s features. Second, the position of each feature is localized. Next, the orientations are assigned based on image gradient directions. The scale-space D(x,y,σ) is computed using a DoG function with the aim of extracting the locations of candidates’ features. The original images are smoothed successively using a variable-scale ($\sigma_{1}$,$\sigma_{2}$, and $\sigma_{3}$) Gaussian function and the scale-space images is calculated by subtracting two successive smoothed images (as shown in Figure 2). *x* and *y* represent the coordinates of the image, while σ is the scale of the Gaussian function.

Lowe’s algorithm has been used in several applications such as multi view matching [49], object tracking [50], etc. Similarly, SIFT has been extensively used within the context of robust image watermarking [41,42].

## 4. Proposed Scheme

In this paper, we propose a hybrid robust image watermarking scheme based on DWT-DCT and SIFT for copyright protection. The main contribution of the proposed method is that it ensures both robustness to signal processing and geometrical attacks using the DWT-DCT domain to embed the watermark and SIFT descriptor, respectively, while preserving the high imperceptibility of the watermarked image. The reason behind using DWT is its excellent spatial localization and multiresolution characteristics, which are similar to the human visual system (HVS) [22], while the choice of using DCT is its strong energy compaction property [23] and good robustness against common image processing attacks. Combining these two well-known transforms, the proposed method can withstand common signal processing manipulations, including filtering, noise, JPEG compression, among others, while ensuring high imperceptibility. Moreover, RST geometric correction using SIFT ensures robustness against geometric attacks.

### 4.1. Embedding Process

**Figure 3:**
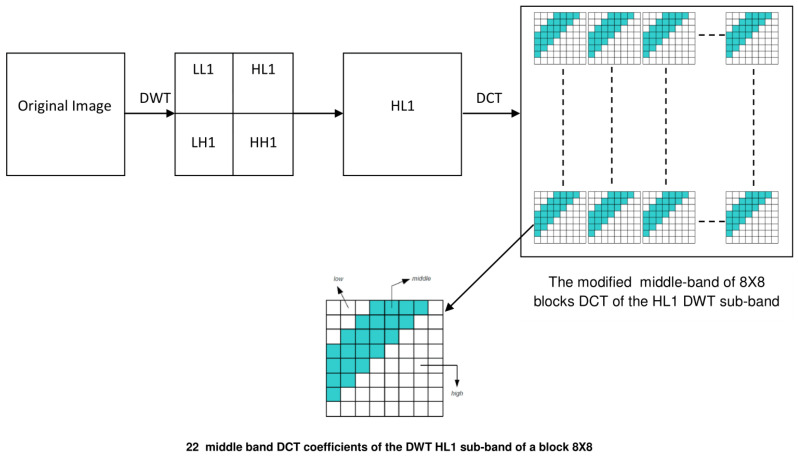
Demonstration of watermark embedding: the watermark is inserted in the 8×8 blocks of the DCT middle bands of the HL1 sub-bands of the DWT.

**Figure 4:**
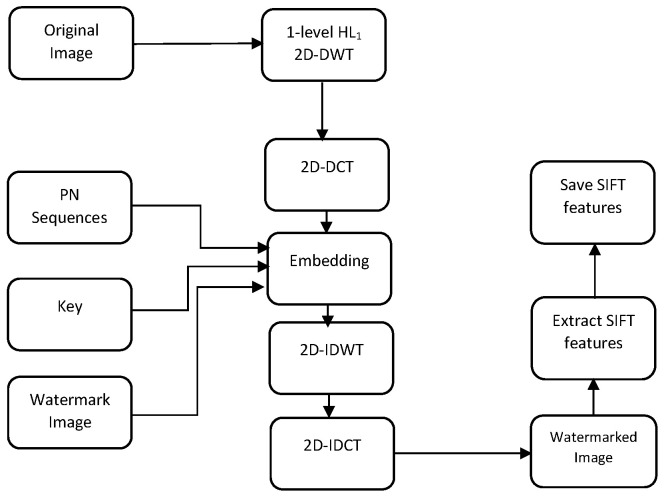
The proposed embedding scheme.

First, the original image is decomposed into four sub-bands LL${}_{1}$, HL${}_{1}$, LH${}_{1}$ and HH${}_{1}$ by performing the 1-level Haar 2D-DWT. Next, the HL${}_{1}$-sub-band is divided into non-overlapping 8×8 blocks and the 2D-DCT is applied to each block. The choice of HL${}_{1}$ has been driven by the fact that this sub-band ensures a good tradeoff between robustness and imperceptibility compared to LL${}_{1}$ and HH${}_{1}$. Afterward, two uncorrelated pseudo-random sequences are generated using a secret key. Each sequence is a vector composed by {−1,1} values with a normal distribution having zero mean and unity variance. The first sequence is for bit 0 (PN${}_{zero}$) while the second one is for bit 1 (PN${}_{one}$). The motivation behind this choice (normally distributed watermark) is the robustness to the attacks trying to produce an unwatermarked document by averaging multiple differently watermarked copies of it [51]. On the detection side, it is important that the PN sequences are statistically independent. This constraint is granted by the pseudo-random nature of the sequences. In addition, such sequences could be easily regenerated by providing the correct seed (key). The used watermark is a binary image. The inserted information is the PN-sequences, according to the watermark bits. If the watermark bits are 0 then the inserted information is (PN${}_{zero}$), otherwise (PN${}_{one}$) is inserted. Gray scale image could be used as watermark. However, the nature of the watermark (binary image, gray-scale image) is not the main concern since the application of the proposed watermarking image scheme is copyright protection. Then, for each block, the two pseudo-random sequences are embedded in the DCT mid-band of the HL${}_{1}$ coefficients according to the watermark bit (shown in blue in Figure 3). The Equation (4) is used to insert the sequence PN${}_{zero}$ if the watermark bit is 0 while Equation (5) is used in the case of bit 1.

(4) $$Y=X+\lambda \times PN_{zero}$$

(5) $$Y=X+\lambda \times PN_{one}$$

where *X* is the original DCT mid-band of the HL${}_{1}$ of the DWT, and *Y* is the modified DCT mid-band of the HL${}_{1}$ of the DWT. λ is the watermark strength that adjusts the tradeoff between imperceptibility and robustness requirements. This parameter is empirically chosen so that it ensures the best tradeoff between robustness and imperceptibility. Note that rather than tuning the parameter λ empirically, it could be statistically tuned based on some optimization criteria such as the error rate, the PSNR, the SSIM, or the normalized correlation. Next, the inverse DCT (2D-IDCT) is carried out for each modified block, and the inverse 2D-DWT (2D-IDWT) is performed to obtain the watermarked image. Finally, SIFT features are extracted and saved in order to correct the geometrical distortions in the extraction process. In most situations of copyright protection applications, the owner of the image is the only one to possess the secret key and the SIFT keypoints needed to extract the watermark. In case there is a need to share with someone else, for each image, the SIFT features and the secret key need to be shared with the extracting side. Therefore, the method is semi-blind since the key points are needed in the extracting process. The proposed watermark embedding is illustrated in Figure 4.

The steps of watermark insertion are described in detail in Algorithm 1.
**Algorithm 1** Watermark embedding**Require:** 
Original image, Watermark, key, PN${}_{zero}$, PN${}_{one}$.**Ensure:** 
Watermarked image, SIFT features.
Perform 1-level Haar 2D-DWT to decompose the original image into four sub-bands LL${}_{1}$, HL${}_{1}$, LH${}_{1}$ and HH${}_{1}$.HL${}_{1}$-sub-band of level-1 is divided into non-overlapping 8×8 blocks.Apply the 2D-DCT to each HL${}_{1}$ block.Generate two uncorrelated pseudo-random sequences using a secret key. One sequence for bit 0 (PN${}_{zero}$) and the second sequence for bit 1 (PN${}_{one}$).For each block, insert the two pseudo-random sequences in the DCT mid-band of the HL${}_{1}$ coefficients according to the watermark bit. If the watermark bit is 0 the Equation (4) is used. The Equation (5) is used otherwise.Apply the inverse DCT (2D-IDCT) for each modified block.Perform the inverse 2D-DWT (2D-IDWT) to obtain the watermarked image.Extract SIFT features and save them.

### 4.2. Extraction Process

The extracting process is divided into two steps: geometrical distortions correction and watermark extraction. Before extracting the watermark, the first step is to correct the geometric manipulations that the attacked image has undergone. To this end, SIFT features are extracted first from the attacked image, and matching is performed between them and the recorded features saved in the insertion step. The idea behind using SIFT relies on the fact that it is RST invariant [52].

It is worth noticing that the proposed method doesn’t require the original image but SIFT features that make it semi-blind. However, the scheme can be blind if no geometric distortions are performed.

In order to correct image rotation attack, the attacked image should be rotated by $R_{c}$. The mathematical formulation of the correction angle is calculated as follows:

(6) $$R_{c}=\frac{1}{N}\sum_{i=1}^{N}\theta_{i}$$

where $\theta_{i}=arccos\frac{\left(\vec{V_{w1}}.\vec{V_{w2}}\right)}{\left|\vec{V_{w1}}\right|\left|\vec{V_{w2}}\right|}$, $\vec{V_{w1}}$ and $\vec{V_{w2}}$ are two vectors composed of two keypoints taken from the watermarked image and the rotated image, respectively. *N* denotes the number of valid matching points. According to Equation (6), the rotation angle is calculated from every two pairs of matching points. Afterward, the angle is corrected by calculating the average sum of angles.

Similarly, to correct the scaling attack, the attacked image should be scaled by $S_{c}$.

(7) $$S_{c}=\frac{1}{N}\sum_{i=1}^{N}\frac{Sw_{i}}{Ss_{i}}$$

where $Sw_{i}$ and $Ss_{i}$ are the scale values of the matching point in watermarked image and scaled image, respectively. Thus, the scaled image can be corrected by scaling it with $S_{c}$.

To correct the translation attack, $CT_{x}$ and $CT_{y}$ are used to correct the translated image on the horizontal and vertical location of coordinates.

(8) $$CT_{x}=\left\{\begin{array}{cc} T_{x}-W_{x}+N & T_{x}<W_{x}\\ T_{x}-W_{x} & otherwise \end{array}\right\}$$

(9) $$CT_{y}=\left\{\begin{array}{cc} T_{y}-W_{y}+N & T_{y}<W_{y}\\ T_{y}-W_{y} & otherwise \end{array}\right\}$$

Thus, after performing the correction of geometric attack step, the weakness against RST attacks can be avoided. The watermark can be extracted perfectly when the watermarked image suffers from this kind of attacks.

The second step is to extract the watermark. To do so, it is sufficient to carry out the 1-level HL${}_{1}$ of the 2D-DWT and calculate the 2D-DCT of the HL${}_{1}$. Afterwards, two pseudo-random sequences using the same key of embedding are generated. Next, the  watermark is extracted by calculating the correlation between the PN sequences and the modified coefficients as shown in Equation (10).

(10) $$W_{i}=\left\{\begin{array}{c} 0\;if\;Corr(0)>Corr(1)\\ 1\;if\;Corr(1)>Corr(0) \end{array}\right\}$$

where Corr(0) is the correlation between the DCT middle frequency of the HL${}_{1}$ coefficients and $PN_{zero}$, and Corr(1) is the correlation between the DCT middle frequency of the HL${}_{1}$ coefficients and $PN_{one}$. Finally, the watermark image is extracted.

**Figure 5:**
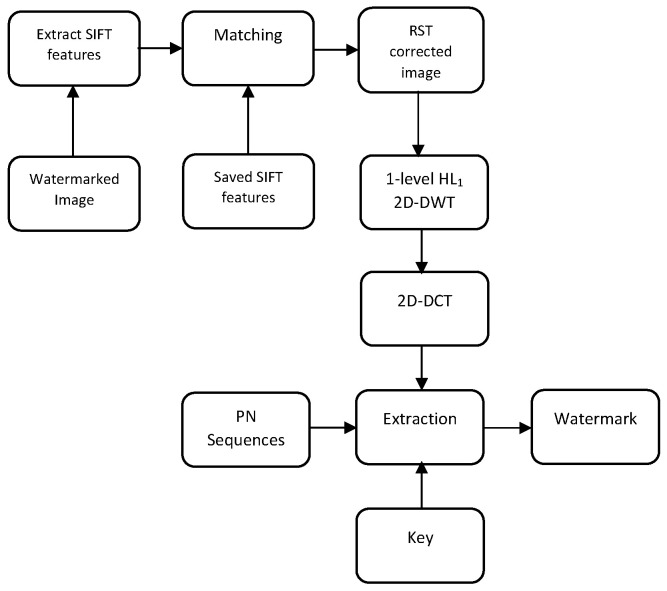
The proposed extracting scheme.

Figure 5 sketches the watermark extracting process that is described in detail in Algorithm 2.
**Algorithm 2** Watermark extracting**Require:** Watermarked image, key, SIFT features.**Ensure:** 
Watermark.
Extract SIFT features from the attacked watermarked image.Feature matching between the extracted SIFT features and the recorded ones in the embedding process.Geometric distortions correction of the image.Perform 1-level HL${}_{1}$ of the 2D-DWT.Apply 2D-DCT to HL${}_{1}$.Generate two pseudo-random sequences using the same key of embedding.Extract the watermark using the correlation between the PN sequences and the altered coefficients as shown in Equation (10).

## 5. Experimental Results

### 5.1. Experimental Setup

The performance of the proposed technique is evaluated on thirty 512×512 standard gray-scale natural images. The images have been carefully selected in order to cover a wide range of images (indoor, outdoor, portrait, etc., texture). These images include the most commonly used images in the watermarking literature. (’Baboon’, ’Pepper’, ’Cameraman’, ’Lena’, ’Goldhill’, ’Walkbridge’, ’Womanblonde’, ’Livingroom’, ’Pirate’ and ’Lake’) and other gray-scale images taken from [53] (Figure A3) in the experiments to assess the imperceptibility and the robustness of the proposed work. A 64×64 binary image was taken as a watermark.

**Figure 6:**
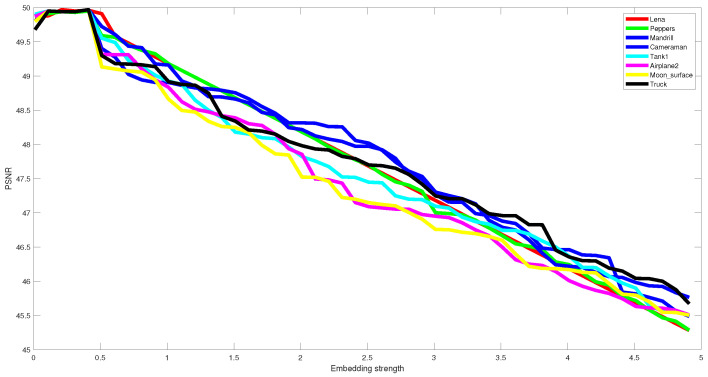
The obtained PSNR values using different embedding strength for several test images.

**Figure 7:**
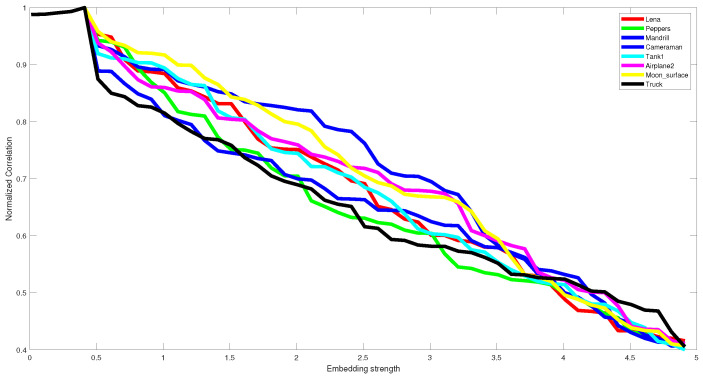
The obtained NC values using different embedding strength for several test images.

The parameter lambda, which denotes the embedding strength of the embedded watermark, affects the visual quality and robustness. This value is chosen in such a way that ensures the best tradeoff between imperceptibility and robustness. To this end, extensive experiments have been conducted using empirically several values, ranging from 0.01 to 5, to determine the value ensuring this tradeoff. This parameter is tuned experimentally, and we kept lambda = 0.4 (see Figure 6 and Figure 7). The same lambda value is used for all the test images. Figure 6 and Figure 7 illustrate the effect of lambda on the performance of the proposed method in terms of imperceptibility and robustness.

### 5.2. Evaluation Measures

#### 5.2.1. Imperceptibility

Subjective evaluation experiments is the gold standard to measure the imperceptibility. However, such a process needs heavy technical and human resources to be conducted [54,55]. This is the reason why the objective metrics are used to assess visual quality of the watermarked images.

In order to evaluate the imperceptibility of the watermarking methods, several metrics have been proposed. Peak signal to noise ratio (PSNR) is the most widely used metric in the watermarking literature to measure the distance between the original image and the watermarked one. It is defined as follows:

(11) $$PSNR=10\log(\frac{MAX^{2}}{MSE})$$

where MAX is the maximum possible pixel value of the image, which is equal to 255 for an 8 bit per pixel representation, and MSE is given by:

(12) $$MSE=\frac{1}{mn}\sum_{i=0}^{m-1}\sum_{j=0}^{n-1}{\left[I\left(i,j\right)-K\left(i,j\right)\right]}^{2}$$

where I(i,j) and K(i,j) refers to the original image and the watermarked image, respectively. *m* and *n* are the dimensions of the image.

The structural similarity (SSIM) index performs similarity measurement using a combination of three heuristic factors that is, luminance comparison, contrast comparison, and structure comparison. It is the most influential perceptual quality metric [56]. It is defined by (13).

(13) $$SSIM\left(I_{0},I_{w}\right)=\frac{\left(2\mu_{I0}\mu_{Iw}+c_{1}\right)\left(2\sigma_{I_{0}I_{W}}+c_{2}\right)}{\left(\mu_{I0}^{2}+\mu_{Iw}^{2}+c_{1}\right)\left(\sigma_{I0}^{2}+\sigma_{Iw}^{2}+c_{2}\right)}$$

where $I_{0}$ and $I_{w}$ are, respectively, the original image and the watermarked image, $\mu_{I0}$ and $\mu_{Iw}$ are, respectively, the local means of $I_{0}$ and $I_{w}$, $\sigma_{I0}^{2}$ is the variance of $I_{0}$ whereas $\sigma_{Iw}^{2}$ is the variance of Iw, $c_{1}$ and $c_{2}$ are two variables used to stabilize the division with weak denominator.

#### 5.2.2. Robustness

The robustness of our work is evaluated using normalized correlation (NC) and bit error rate (BER) between the original watermark and the extracted one.

The normalized correlation (NC) is a widely used attribute for quantifying the robustness of the underlying watermarking technique against various attacks. It measures the similarity between the extracted watermark and the original watermark. It is defined by:

(14) $$NC=\frac{\sum_{i=1}^{M}\sum_{j=1}^{N}{\left[\begin{array}{cc} W(i,j) & \times W^{\prime }\left(i,j\right) \end{array}\right]}^{2}}{\left(\begin{array}{cc} \sqrt{\sum_{i=1}^{P}\sum_{j=1}^{Q}{\left[W(i,j)\right]}^{2}} & \sqrt{\sum_{i=1}^{P}\sum_{j=1}^{Q}{\left[W^{\prime }\left(i,j\right)\right]}^{2}} \end{array}\right)}$$

where *W* and $W^{\prime }$ are the original and the extracted watermark, respectively.

To further evaluate the robustness of the proposed work, bit error rate (BER) is used to calculate the bit error rate between the original watermark and the extracted one. It is defined as follows:

(15) $$BER=\sum_{i=1}^{m}\sum_{j=1}^{n}\frac{W_{i,j}⨁W_{i,j}^{\prime }}{\left(m\times n\right)}$$

where $W_{i,j}$ and $W_{i,j}^{\prime }$ are original and extracted watermark with size of (m×n) and ⨁ refers to X or operation.

### 5.3. Evaluation of Imperceptibility

**Figure 8:**
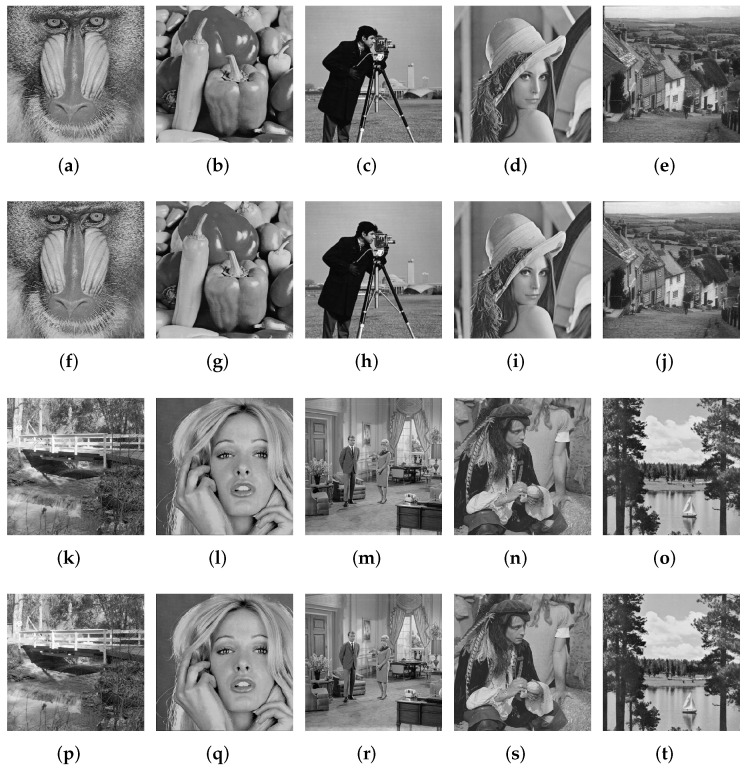
The original images and their corresponding watermarked one. Original images: (**a**) Mandril, (**b**) Peppers, (**c**) Cameraman, (**d**) Lena, (**e**) Goldhill, (**k**) Walkbridge, (**l**) Woman blonde (**m**) Livingroom, (**n**) Pirate, (**o**) Lake. Watermarked images: (**f**) Mandril, (**g**) Peppers, (**h**) Cameraman, (**i**) Lena, (**j**) Goldhill, (**p**) Walkbridge, (**q**) Woman blonde, (**r**) Livingroom, (**s**) Pirate, (**t**) Lake.

**Figure 9:**
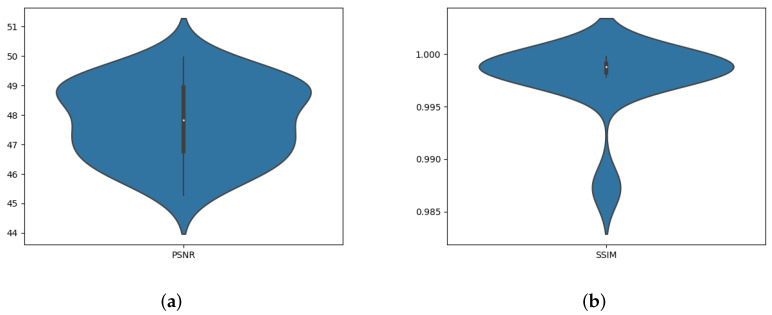
The violin plot of all 30 test images: (**a**) Violin plot of the PSNR calculated between original and watermarked images, (**b**) Violin plot of the SSIM calculated between original and watermarked images.

Table 1 exhibits the imperceptibility of the proposed technique measured by the two well-known metrics PSNR and SSIM for all test images and their average. One can notice that the proposed method can ensure good imperceptibility according to the obtained values of PSNR and SSIM in Table 1, Figure 6, Figure 8 and Figure 9. We believe that the main reason stands on the fact that the watermark is embedded in the middle DCT coefficients of the LH DWT sub-band that ensures high imperceptibility.

One can remark from Table 1 that the imperceptibility of the proposed scheme is insensitive to the image nature. Figure 8 and Figure A3 show the original images and their corresponding watermarked ones. Moreover, as depicted in Figure 8 there is no visual distortion between the original images and the watermarked ones.

The violin plot representation of PSNR and SSIM of the proposed scheme. The values of PSNR and SSIM shown in Table 1 are represented in black in Figure 9. In addition, according to Figure 9, the majority of SSIM values are concentrated between 0.995 and 0.9998. PSNR values are between 45.28 and 49.97, illustrating the good robustness of the proposed scheme regardless of the image nature.

### 5.4. Evaluation of Robustness

**Figure 10:**
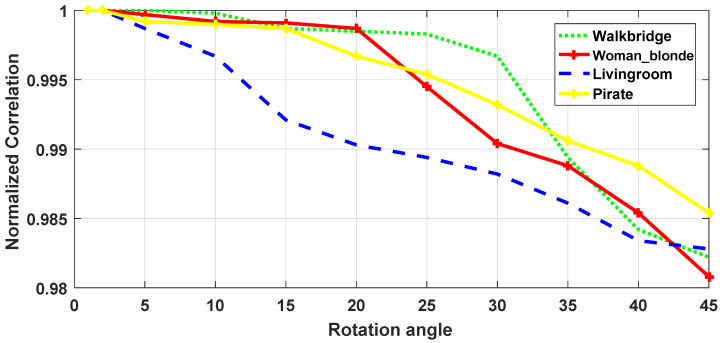
Robustness in terms of NC against rotation attack.

**Figure 11:**
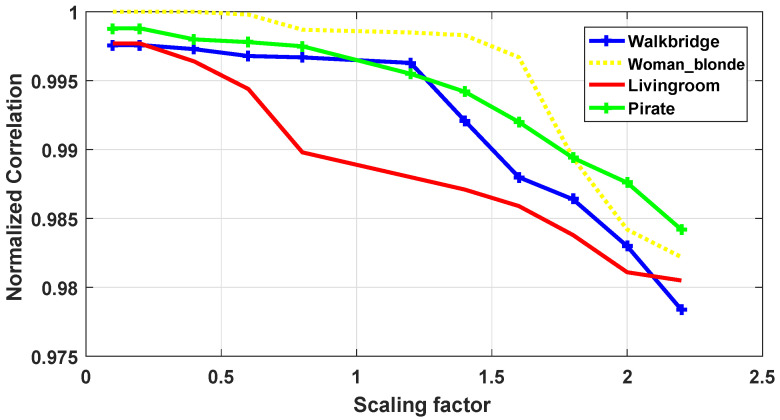
Robustness in terms of NC against scaling attack.

**Figure 12:**
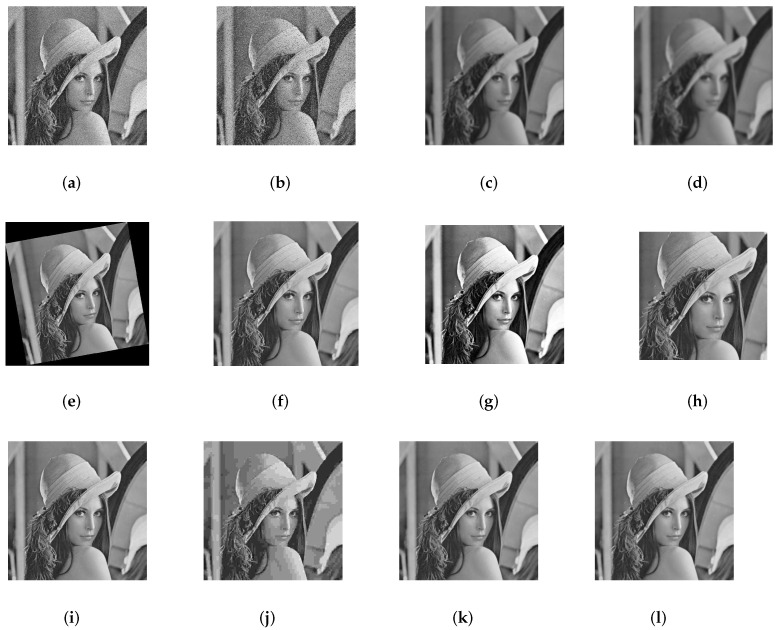
Sample of attacked watermarked images: (**a**) Gaussian noise addition with zero mean and standard deviation (0.01), (**b**) Salt and pepper noise with noise density 0.01, (**c**) Low-pass Gaussian filtering (σ=0.5,7×7), (**d**) Low-pass Gaussian filtering (σ=0.6,7×7), (**e**) Rotation (10°), (**f**) Scaling (1.2), (**g**) Histogram equalization, (**h**) Cropping(25%), (**i**) JPEG compression with quality factor 60, (**j**) JPEG compression with quality factor 5, (**k**) Gaussian smoothing (σ=0.7,7×7), (**l**) Gaussian smoothing (σ=0.9,7×7).

Since the application of the proposed scheme is copyright protection, robustness is the most important requirement. Image processing, JPEG compression and geometrical manipulations are the three categories of attacks that watermarked images have undergone. Image processing attacks include Gaussian noise (GN), salt and pepper noise (SPN), low-pass Gaussian filtering (LPGF), histogram equalization (HE), Gaussian smoothing (GS), median filtering (MF). JPEG compression and JPEG2000 represent the compression attacks. Rotation (ROT), scaling (SC), translation (TR), and cropping (CR) were selected as geometrical attacks. Figure 10 and Figure 11 show the robustness of the proposed method in terms of NC against rotation and scaling using four test images with several textures. Figure 12 depicts Lena image after performing several attacks. The false alarm probability is not discussed in the paper, and the robustness of our work is evaluated using normalized correlation (NC) and bit error rate (BER) between the original watermark and the extracted one.

The 30 test images used in the experiments were chosen according to their characteristics (texture, indoor, outdoor, etc). In addition, some typical images have been used in the experiments. The images used in Figure 8 and Figure 10 are selected in such a way that they represent differences in these characteristics.

Experiments were performed to evaluate the limitations of the proposed method. The parameter values of the attacks have been tuned such that the watermark is no longer recovered. We consider that with an NC value lower than 0.7, distortions are sufficiently high such that the watermark cannot be recovered.

**Figure 13:**
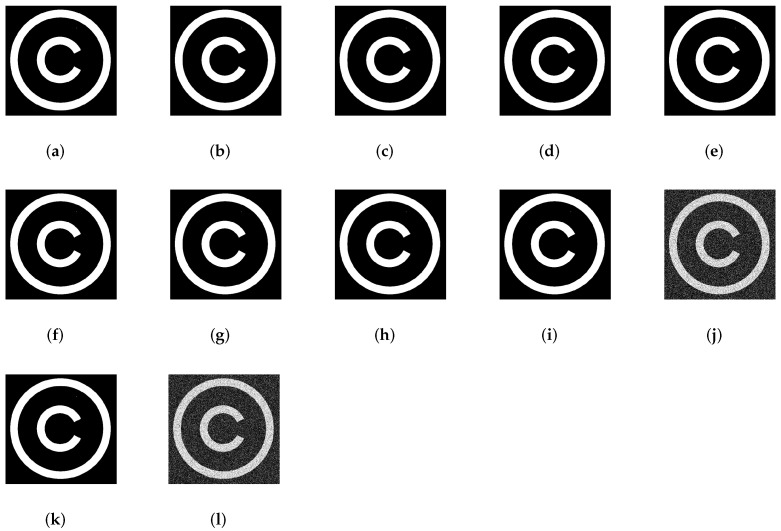
Extracted watermarks after: (**a**) Gaussian noise addition with zero mean and standard deviation (0.01), (**b**) Salt & pepper noise with noise density 0.01, (**c**) Low-pass Gaussian filtering (σ=0.5,7×7), (**d**) Low-pass Gaussian filtering (σ=0.6,7×7), (**e**) Rotation (10°), (**f**) Scaling (1.2), (**g**) Histogram equalization, (**h**) Cropping( 25%), (**i**) JPEG compression with quality factor 60, (**j**) JPEG compression with quality factor 5, (**k**) Gaussian smoothing (σ=0.7,7×7), (**l**) Gaussian smoothing (σ=0.9,7×7).

Figure 13 displays the extracted watermarks after different attacks, including histogram equalization, JPEG compression, salt and pepper noise, Gaussian noise, cropping, rotation, etc. It can be observed that although the watermarked images have been exposed to these attacks, the watermark is almost extracted perfectly.

Table 2 shows the robustness in terms of NC for several images against Gaussian noise using zero mean, 0.001 and 0.01 variances, respectively, and the NC average for 30 test images. Table 3 shows the obtained NC values after carrying out salt and pepper noise with zero mean and 0.001 and 0.01 variances and the NC average for 30 test images. According to Table 2 and Table 3, the NC values are above 0.99 for 30 images. However, for both Gaussian and salt and pepper noise, when the variance is 0.01, the obtained NC values for Mandrill are below 0.99 (NC = 0.9865 and 0.9898, respectively). In addition, the obtained average values of NC, shown in Table 2 and Table 3 for both attacks are above 0.99 for the 30 test images.

Gaussian smoothing is a very common operation in image processing [57]. It consists of removing detail and noise [58]. The Gaussian smoothing has been applied to the test images with different standard deviations and window sizes. As depicted in Table 4, the proposed technique is able to withstand Gaussian smoothing attack. Even with a standard deviation σ=0.9 and 7×7 window size, the obtained NC values are greater than 0.96. It can be noticed from Table A8 that the proposed technique can withstand Gaussian smoothing for all thirty test images.

The low-pass Gaussian filtering attack is also one of the common manipulations in image processing. It aims to remove high-frequency components from the image. The watermarked images are filtered with a low-pass Gaussian filter using several mask sizes (3×3), (5×5), and (7×7) and two standard deviation values (σ=0.5, σ=0.6). It can be concluded from Table 5 that high NC values are achieved under the low-pass Gaussian filtering with the different mask sizes. In addition, one can see from Table A8 that the proposed method can resist low-pass Gaussian filtering for the dataset, and the minimum average NC value is 0.9812.

Robustness against lossy compression is crucial due to the wide diffusion of lossy compression tools and the huge use of this image format. To assess the performance from this point of view, JPEG compression is iteratively applied to the watermarked images, each time decreasing the quality factor, ranging from 90 to 5. Table 6 summarizes the results obtained in terms of NC after JPEG compression using several quality factors for the 30 test images. As can be seen, the proposed method exhibits good robustness against this attack. Furthermore, the robustness against JPEG2000 has been investigated using different compression ratios (CR) varying from 1 to 10. Table 7 depicts the results in terms of normalized correlation against JPEG2000 attack using 30 images. It can be seen from Table 6 that the proposed method can withstand JPEG attack when the quality factor is above 40. For quality factors below 40, the watermark can be well recognized since the NC values are above 0.7. Regarding JPEG2000 compression, it can be seen from Table 7 that the proposed method can resist to JPEG2000 attack when the compression ratio (CR) is below 10. We consider that the obtained results are comparable since the minimum NC average of all test images is above 0.7.

One can see from Table 7 that the proposed method is quite robust against JPEG2000. The proposed method shows its limitation when the compression ratio (CR) is larger than 6 but the results are still satisfactory (NC =0.7031, CR=10).

**Figure A1:**
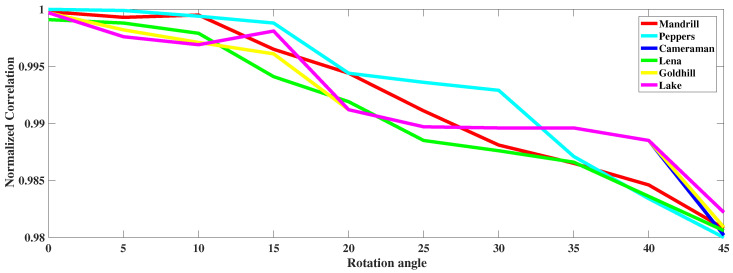
Robustness in terms of NC against rotation attack for several test images.

**Figure A2:**
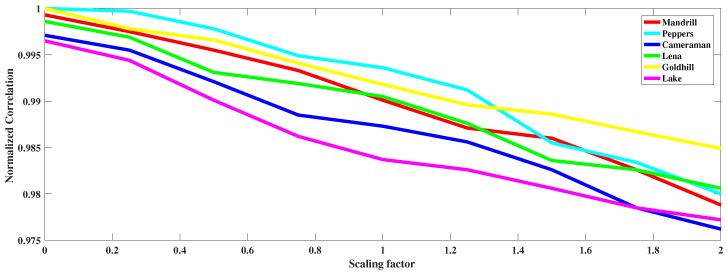
Robustness in terms of NC against scaling attack for several test images.

Figure 10 and Figure 11 show the robustness in terms of NC of the proposed technique against rotation and scaling using four test images with several textures, respectively. The rotation attack is applied using several rotation angles ranging from 1 to 45. The obtained results presented in Table 8, show the good robustness of the proposed method against rotation attack. Similarly, the test images have undergone scaling attack with different scaling factors ranging from 0.1 to 2.5. It can be seen from Figure 11 that the proposed technique is able to withstand scaling attack for all images. We note that the results for selected images under test are reported in Figure 10 and Figure 11. The remaining results for rotation and scaling attacks are reported in Figure A1 and Figure A2 and Table A8. It can be observed from Table A8 that the proposed method is robust to rotation and scaling attacks for all the thirty test images. The average NC values are below 0.9877 and 0.9872 for rotation and scaling, respectively.

To further test the robustness of the proposed method, different combinations of attacks have been carried out. Table 9 sketches a set of combinations of image processing attacks, while Table 10 exhibits a set of combinations of both geometric and image processing attacks. It can be concluded from these tables that the proposed method is robust to attack combination for the both types of attacks since all the obtained NC values are greater than 0.96. In addition, one can see from Table A8 the resistance to combined attacks of the proposed method for all the thirty test images.

Moreover, as depicted in Figure 13, the extracted watermark is well recognizable even after applying several attacks to the watermarked image which indicates the good robustness of the proposed method.

**Figure A3:**
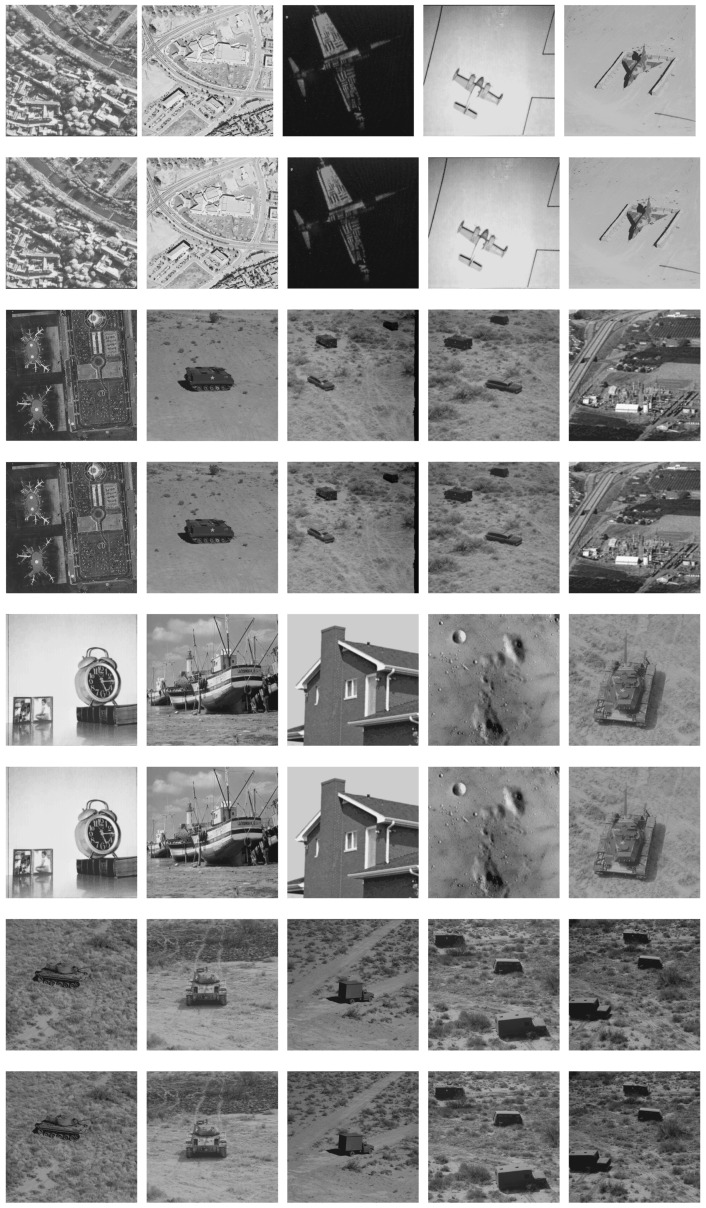
Twenty test images used in the experiments taken from [53] and their corresponding watermarked images. Odd lines represents the original images and the even lines refer to the corresponding watermarked images.

Table A7 shows the robustness evaluation using bit error rate (BER) against a wide range of attacks. The presented results represent the average values of BER for 30 test images shown in Figure 8 and Figure A3. It can be seen from Table A7 that the proposed method can resist the majority of the attacks such as image processing (filtering, noise, etc.), JPEG compressions (JPEG and JPEG2000), geometric attacks (rotation, scaling, translation, and cropping) and combined attacks. The obtained values of BER calculated between the original watermark and the extracted one are near zero, which illustrates the robustness of the proposed technique.

One can see from Table A7 that the robustness performance in terms of BER decreases when the quality factor of JPEG decreases. However, even for high values of this parameter (5%), the watermark can still be recovered. Similarly, when the noise is applied with a high density (0.01 or higher), the BER increases. However, for Gaussian smoothing for 7×7 and σ=9, the obtained results are comparable.

It can be observed from Table A7 that the proposed method is robust against histogram equalization, cropping, and scaling. As shown in Table A7, the proposed technique can resist to rotation for the angles below 40°, Gaussian noise (σ=0.005, salt and pepper noise (σ=0.001)), Low-pass Gaussian filtering for (σ=0.5,(3×3,5×5,7×7,9×9) and σ= 0.6 (3×3)), Gaussian smoothing (σ=0.5,5×5), JPEG when quality factor is above 50%, and JPEG2000 for compression ratio below 8. According to Table A7, it can be seen that the robustness of the proposed method has its limitations for the following attacks parameters: Low-pass Gaussian filtering for (σ=0.6,(5×5,7×7,9×9)), Gaussian smoothing (σ=0.5,(5×5,7×7,9×9)), JPEG when quality factor is above 50%, and JPEG2000 for compression ratio below 8.

Table A8 reports the robustness results in terms of NC with the aim of evaluating the limitations of the proposed method. For Gaussian noise until the density reaches the value 0.8, one can still recover the watermark. For salt & pepper noise with density 0.7, the watermark can be extracted. Regarding JPEG compression with quality factor below 4, the extracted watermark cannot be appropriately extracted. After applying the Gaussian smoothing with a window of 9×9 and σ=10), the extracted watermark cannot be recognized. To summarize, one can see from Table A8 that the proposed method can’t resist these attacks. This is due to the high damage caused by these severe attacks which cause the huge decrease of robustness in terms of NC.

### 5.5. Comparison with Alternative Methods

To show the competitiveness of our approach, we compare it with our previous work [24] as well as schemes in [13,21,26,27,28,29,34,44,45] in terms of imperceptibility and robustness.

We note that all the test images are under the same attack in Table 2, Table 3, Table 8, Table A7 and Table A8.

#### Comparison of Imperceptibility and Robustness

In Table 11, is presented the comparison in terms of imperceptibility between the proposed scheme and the schemes in [26,27,28]. It is clear from Table 11 that the proposed method shows better imperceptibility compared with Lagzian et al. [26], Makbol et al. [27] and Singh et al. [28] methods in terms of PSNR.

It can be seen from Table 12 that the use of DWT-DCT only fails to provide robustness to geometric attacks, while using SIFT avoids weakness against this kind of attack.

We have compared our work with state-of-the-art methods based on the presented results of the latter. We have not implemented alternative methods. Thus, for the results presented in Table 13, Table 14, Table 15, Table 16 and Table 17, Table A1 and Table A2, we have compared the proposed work only for the attacks exhibited in the alternative techniques.

To further evaluate the robustness of the proposed method, it has been compared to [13,21,26,27,28,29] in terms of normalized correlation (NC). Additionally, the watermarked images have undergone several combined attacks. These combined attacks include image processing manipulations as well as geometric operations.

Table 13 and Table 14 show the comparison results in terms of robustness with [26,27] methods under several attacks including, rotation, Gaussian noise, salt and pepper noise, median filtering, JPEG compression, histogram equalization. It can be observed that our method outperforms the schemes in [26,27] in the majority of the attacks. Table 15 depicts the robustness results in terms of NC against different attacks compared to Singh et al. method [28]. One can see from Table 15 that the proposed method shows high robustness compared to [28] against different attacks including Gaussian noise, salt and pepper noise, median filtering, histogram equalization, JPEG compression, and rotation.

To further evaluate the robustness performance of the proposed method, we compare it with Zhang et al.’s method [13]. To this end, the watermarked image has undergone several geometric distortions as well as image processing attacks. The obtained results, shown in Table 16, indicate the superiority of the proposed scheme. The rotation and scaling attacks have been investigated in Table 14, Table 15, Table 16 and Table 17, respectively. It can be seen from these tables that the proposed method is quite robust to rotation and scaling attacks for several test images thanks to the SIFT operator. In addition, the proposed technique outperforms the schemes in [13,21,27,28].

Table 17 shows that one can distinguish three categories of attacks. In the first category, including Gaussian noise, salt and pepper noise, and cropping attacks, the proposed method clearly outperforms [13]. For JPEG, rotation and scaling attacks results are quite comparable, even if the proposed method performs slightly better. Finally, the third category contains a single attack (median filtering). In this case, [13] outperforms the proposed method.

**Figure 14:**
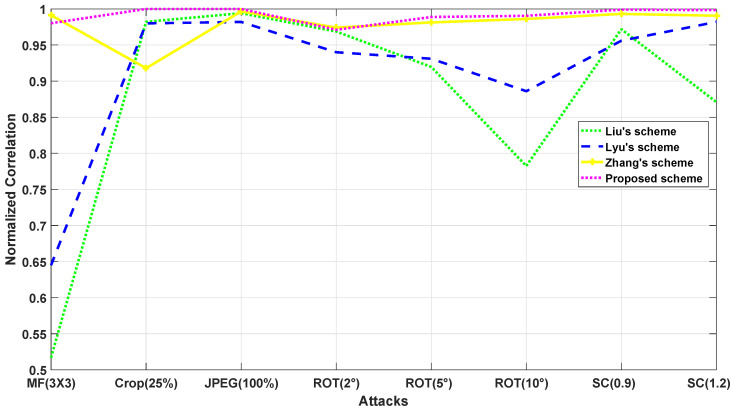
Robustness comparison in terms of NC between Liu et al.’s scheme [21], Lyu et al.’s scheme [29], zhang et al.’s scheme [13] and the proposed scheme.

As shown in Table 17 the proposed method is quite robust to cropping, median filtering, rotation, scaling and outperforms the methods in [13,21]. Table 9 shows the obtained results in terms of NC after carrying out several combined attacks. It can be concluded from Table 9 that the proposed method is able to withstand combined attacks (all NC values are above 0.9937). Moreover, our scheme shows high robustness compared to Zhang’s scheme [13]. Figure 14 displays the robustness comparison results in terms of normalized correlation between zhang’s scheme [13], Lyu’s scheme [29], Liu’s scheme [21] and the proposed scheme. Comparison with Luy et al. method [29], (blue curve in Figure 14) shows that the proposed method is more effective whatever the attack under test. Note that it uses a single transform with SIFT descriptor. This highlights the importance of using both transforms. A deeper look shows that it performs particularly less for small rotation (5° and 10°) and scaling. The differences between the two methods are less pronounced for JPEG, cropping, and very small rotation (2°).

Regarding the comparison with Liu et al. method [21] (green curve in Figure 14), it appears that the proposed technique is quite superior for median filtering attack. Indeed the NC values drop from 0.98 to 0.5. In addition, the proposed method shows superior robustness for small rotation (5° and 10°) and scaling. The results are comparable for JPEG, cropping, and minimal rotation (2°) attacks. This corroborates the reported properties of the SVD in cases where perturbations are small [12]. Figure 14 shows that the method presented in [13] (yellow curve) gives comparable results in terms of NC for JPEG, rotation, and scaling attacks compared to the proposed method.

For cropping, the proposed method exhibits higher robustness as compared to the scheme reported in [13], while this is the contrary for median filtering attack. These results are not surprising. Indeed, both methods use two transforms associated with SIFT descriptor.

One can see from Table 13 that the proposed method outperforms the technique proposed in [26] in all attacks except for JPEG compression with quality factor 50 and median filtering. For these two attacks even the method in [26] outperforms the proposed method, the robustness results in terms of NC are comparable. It can be seen from Table 14 that the proposed method is robust against the tested attacks except for JPEG compression in which the alternative method [27] shows its superiority in terms of robustness. Similarly, in Table 15, Table 16 and Table 17, it can be observed that the proposed method fails to show its superiority in terms of robustness in only one case (median filtering (in Table 15, Table 16 and Table 17), and rotation (in Table 17)).

It can be seen that the proposed technique outperforms the scheme in [29] for a wide range of attacks such as, rotation, JPEG, salt and pepper noise, median filtering, and cropping (25% and 50%). One can see from Table A2 that the proposed method can obtain comparable results in terms of robustness for centered cropping (75%).

The robustness of the proposed method is compared to our previous work [24] and the scheme in [34]. The attacks used for the comparison are applied to three images (Lena, Peppers, and Baboon) as shown in Table A5 and Table A6. For the three images (Lena, Peppers, and Baboon), the proposed method outperforms the scheme in [34] for JPEG, JPEG2000, histogram equalization, and cropping attacks. In addition, the proposed technique provides the highest robustness performance than the scheme in [24] for JPEG, JPEG2000, histogram equalization, and cropping attacks. In sum, the proposed method shows comparable results in terms of robustness against geometric attacks. At the same time, it can outperform our previous method [24] since the SIFT is used to correct geometric attacks.

Table A3 sketches the comparison of the robustness of the proposed technique with the scheme in [44] after applying several attacks, such as additive noise, median filtering, histogram equalization, JPEG, rotation, and scaling. It can be observed from Table A3 that the proposed algorithm shows high robustness for Gaussian noise, histogram equalization, rotation, and JPEG (when QF is great than 50) attacks. The proposed technique achieves comparable results for median filtering, jpeg when QF is below 40, and scaling (for zoom greater than 0.9) attacks.

Table A4 shows the results of robustness compared to the scheme of Chen et al. [45] in terms of BER. The comparison has been made for three different images Lena, Mandrill, and Peppers. According to Table A4, it can be seen that the proposed method provides high robustness for JPEG, scaling, and median filtering. For rotation and cropping attacks, the proposed method can achieve comparable results in terms of robustness.

To summarize, one can conclude from the experiments that combining a hybrid scheme with SIFT descriptor allows significant gains for several attacks while preserving good imperceptibility.

## 6. Conclusions

In this paper, a robust image watermarking method based on SIFT in the DWT-DCT domain is presented. Its goal is to ensure both robustness against geometric and image processing attacks while preserving high imperceptibility. The proposed method takes the advantages of combining the DWT and DCT transforms to ensure robustness in the face of common image processing attacks such as filtering, histogram equalization, JPEG compression, and noise attacks without degrading the image quality. At the same time, SIFT descriptor characteristics are used to obtain robustness against geometrical attacks, especially rotation, scaling, and translation. The experimental results and comparisons have demonstrated the high robustness of the proposed method for both common image processing attacks and geometrical attacks while preserving a good imperceptibility. Future work will focus on using a meta-heuristic algorithm to find the optimal watermark strength.

## Figures and Tables

**Table 1:** Watermark imperceptibility measured in terms of PSNR (dB) and SSIM.

Test Image	PSNR	SSIM
Mandrill	45.28	0.9865
Lena	48.97	0.9998
Peppers	49.97	0.9988
Cameraman	47.54	0.9987
Goldhill	49.37	0.9998
Walkbridge	46.24	0.9983
Womanblonde	46.57	0.9978
Livingroom	48.64	0.9986
Pirate	47.33	0.9980
Lake	48.11	0.9982
Aerial1	46.31	0.9985
Aerial2	45.88	0.9877
Airplane_U_2	47.23	0.9978
Airplane1	46.75	0.9987
Airplane2	49.53	0.9998
Airport1	49.08	0.9990
APC	48.91	0.9991
Car_and_APCs1	47.44	0.9989
Car_and_APCs2	48.17	0.9989
Chemical_plant	48.62	0.9988
Clock	49.14	0.9992
Fishing_Boat	47.49	0.9989
house	45.86	0.9876
Moon_surface	46.77	0.9988
Tank1	46.33	0.9985
Tank2	47.31	0.9979
Tank3	48.95	0.9991
Truck	48.28	0.9990
Truck_and_APCs	49.55	0.9997
Truck_and_APCs2	48.72	0.9991
Average	47.81	0.9976

**Table 2:** Watermark robustness measured in terms of NC against Gaussian noise for several test images.

Test Image	(μ=0,σ=0.001)	(μ=0,σ=0.01)
Mandrill	1.0	0.9865
Lena	1.0	0.9998
Peppers	0.9998	0.9987
Cameraman	1.0	0.9996
Goldhill	0.9999	0.9998
Walkbridge	1.0	0.9982
Womanblonde	0.9999	0.9978
Livingroom	1.0	0.9986
Pirate	0.9998	0.9980
Lake	1.0	0.9982
Aerial1	0.9982	0.9975
Aerial2	0.9908	0.9878
Airplane_U_2	0.9995	0.9980
Airplane1	0.9992	0.9986
Airplane2	1.0	0.9998
Airport1	1.0	0.9991
APC	0.9996	0.9992
Car_and_APCs1	0.9991	0.9983
Car_and_APCs2	0.9999	0.9985
Chemical_plant	0.9999	0.9991
Clock	1.0	0.9994
Fishing_Boat	0.9990	0.9979
house	0.9912	0.9882
Moon_surface	0.9993	0.9984
Tank1	0.9993	0.9987
Tank2	0.9992	0.9985
Tank3	0.9998	0.9991
Truck	1.0	0.9994
Truck_and_APCs	1.0	0.9995
Truck_and_APCs2	0.9999	0.9992
Average	0.9991	0.9976

**Table 3:** Watermark robustness measured in terms of NC against salt and pepper noise for several test images.

Test Image	(μ=0,σ=0.001)	(μ=0,σ=0.01)
Mandrill	1.0	0.9898
Lena	1.0	0.9998
Peppers	1.0	0.9984
Cameraman	1.0	0.9987
Goldhill	1.0	0.9998
Walkbridge	1.0	0.9983
Womanblonde	0.9996	0.9985
Livingroom	1.0	0.9989
Pirate	0.9999	0.9996
Lake	0.9999	0.9993
Aerial1	0.9986	0.9979
Aerial2	0.9918	0.9879
Airplane_U_2	0.9995	0.9982
Airplane1	0.9995	0.9984
Airplane2	1.0	0.9997
Airport1	1.0	0.9990
APC	0.9995	0.9993
Car_and_APCs1	0.9993	0.9987
Car_and_APCs2	1.0	0.9985
Chemical_plant	1.0	0.9992
Clock	1.0	0.9993
Fishing_Boat	0.9992	0.9983
house	0.9923	0.9881
Moon_surface	0.9994	0.9985
Tank1	0.9994	0.9986
Tank2	0.9993	0.9988
Tank3	0.9996	0.9990
Truck	1.0	0.9994
Truck_and_APCs	1.0	0.9996
Truck_and_APCs2	1.0	0.9994
Average	0.9992	0.9975

**Table 4:** Robustness evaluation measured in terms of normalized correlation after Gaussian smoothing for Mandrill image.

Gaussian Smoothing	Normalized Correlation
3×3	1.0
(σ=0.5) 5×5	1.0
7×7	1.0
3×3	1.0
(σ=0.6) 5×5	1.0
7×7	1.0
3×3	0.9984
(σ=0.7) 5×5	0.9962
7×7	0.9945
3×3	0.9939
(σ=0.8) 5×5	0.9917
7×7	0.9901
3×3	0.9894
(σ=0.9) 5×5	0.9788
7×7	0.9681

**Table 5:** Robustness evaluation measured in terms of normalized correlation after low-pass Gaussian filtering for Mandrill image.

LPGF	Normalized Correlation
3×3	1.0
(σ=0.5) 5×5	1.0
7×7	1.0
3×3	0.9992
(σ=0.6) 5×5	0.9986
7×7	0.9975

**Table 6:** Watermark robustness in terms of NC after JPEG compression for 30 test images.

Quality	Normalized Correlation
90	1.0
80	0.9997
70	0.9918
60	0.9825
50	0.9785
40	0.8981
30	0.7710
20	0.7674
10	0.7355
5	0.7182

**Table 7:** Watermark robustness against JPEG2000 attack in terms of NC for 30 test images.

Compression Ratio	Normalized Correlation
CR=2	1.0
CR=4	0.9913
CR=6	0.9137
CR=8	0.8752
CR=10	0.7031

**Table 8:** Robustness after rotation attack applied to thirty test images.

	Rotation			
**Image**	10°	20°	30°	45°
Mandrill	0.9998	0.9944	0.9877	0.9816
Lena	0.9995	0.9994	0.9987	0.9903
Peppers	0.9997	0.9945	0.9928	0.9802
Cameraman	0.9905	0.9908	0.9912	0.9864
Goldhill	0.9976	0.9913	0.9877	0.9820
Walkbridge	0.9999	0.9965	0.9958	0.9901
Womanblonde	0.9991	0.9982	0.9905	0.9856
Livingroom	0.9979	0.9956	0.9937	0.9869
Pirate	0.9993	0.9991	0.9951	0.9906
Lake	0.9973	0.9914	0.9911	0.9822
Aerial1	0.9992	0.9976	0.9927	0.9857
Aerial2	0.9995	0.9990	0.9954	0.9868
Airplane_U_2	0.9993	0.9987	0.9972	0.9861
Airplane1	0.9997	0.9983	0.9972	0.9842
Airplane2	0.9995	0.9984	0.9956	0.9905
Airport1	0.9998	0.9992	0.9931	0.9879
APC	0.9997	0.9988	0.9928	0.9907
Car_and_APCs1	0.9996	0.9986	0.9937	0.9834
Car_and_APCs2	0.9995	0.9987	0.9936	0.9883
Clock	0.9999	0.9983	0.9958	0.9861
Fishing_Boat	0.9993	0.9981	0.9967	0.9905
House	0.9995	0.9980	0.9976	0.9874
Moon_surface	0.9991	0.9987	0.9945	0.9867
Tank1	0.9994	0.9985	0.9980	0.9904
Tank2	0.9992	0.9984	0.9975	0.9865
Tank3	0.9997	0.9986	0.9972	0.9906
Truck	0.9996	0.9983	0.9976	0.9887
Truck_and_APCs	0.9994	0.9987	0.9961	0.9905
Truck_and_APCs2	0.9993	0.9990	0.9968	0.9861

**Table 9:** Watermark robustness comparison in terms of NC against combined attacks.

Attack	[13]	Proposed Method
Rotation (10°) + JPEG(100)	0.9964	0.9971
Rotation (10°) + GN(0,0.005)	0.9644	0.9952
Rotation (10°) + SPN(0,0.005)	0.9779	0.9951
Rotation (10°) + center cropping(25%)	0.9098	0.9963
Scaling (0.5) + JPEG(100)	0.9920	0.9994
Scaling (0.5) + GN(0,0.005)	0.9239	0.9993
Scaling (0.5) + SPN(0,0.005)	0.9348	0.9992
Scaling (0.5) + center cropping(25%)	0.8807	0.9993
Horizontal translation + JPEG (100)	0.9966	0.9984
Horizontal translation + GN (0,0.005)	0.9190	0.9981
Horizontal translation + SPN (0,0.005)	0.9455	0.9979
Horizontal translation + center cropping (25%)	0.8981	0.9985
Rotation (10°) + Scaling(0.5)	0.9857	0.9945
Scaling (0.5) + Horizontal translation	0.9912	0.9944
Rotation (10°) + Horizontal translation	0.9851	0.9937

**Table 10:** Watermark robustness in terms of NC against combined attacks for Lena.

Attack	NC
HE + GN (0,0.001)	1.0
HE + GN (0,0.01)	1.0
HE + SPN (0,0.001)	1.0
HE + SPN (0,0.01)	0.9834
GN (0,0.001) + JPEG 90	0.9999
GN (0,0.001) + JPEG 70	0.9832
SPN (0,0.01) + JPEG 90	0.9999
SPN (0,0.01) + JPEG 70	0.9832
LPGF (σ=0.5,9×9) + GN (0,0.001)	0.9881
LPGF (σ=0.5,9×9) + GN (0,0.01)	0.9733
LPGF (σ=0.6,9×9) + GN (0,0.001)	0.9810
LPGF (σ=0.6,9×9) + GN (0,0.01)	0.9758
LPGF (σ=0.5,9×9) + SPN (0,0.001)	0.9732
LPGF (σ=0.5,9×9) + SPN (0,0.01)	0.9665
LPGF (σ=0.6,9×9) + SPN (0,0.001)	0.9644
LPGF (σ=0.6,9×9) + SPN (0,0.01)	0.9602
GN (0,0.001) + HE + JPEG 90	0.9999
SPN (0,0.01) + HE + JPEG 70	0.9991
GN (0,0.001) + ROT (15) + HE	0.9996
SPN (0,0.01) + SC (0.8) + HE	0.9993
JPEG 70 + ROT (30) + SC (1.2)	0.9995
JPEG 50 + MF (3×3) + SC (1.2)	0.9627
CR (50%) + ROT (45) + HE	0.9732
JPEG 40 + ROT (30) + SC (0.8)	0.9901
JPEG 60 + MF (3×3) + SC (1.2)	0.9788
CR (50%) + ROT (45) + SC (0.5)	0.9604

**Table 11:** Imperceptibility comparison values in terms of PSNR (db) and SSIM for Lena image of Lagzian et al. [26], Makbol et al. [27], Singh et al. [28], Chen et al. [45] and the proposed method using a watermark of size 256×256.

	Lagzian et al. [26]	Makbol et al. [27]	Singh et al. [28]	Proposed Scheme
PSNR	37.52	43.6769	44.40	48.97
SSIM	0.9865	0.9872	0.9935	0.9998

**Table 12:** Watermark robustness measured in terms of NC with and without SIFT for cameraman image.

Attacks	DWT-DCT	DWT-DCT-SIFT
Rotation		
2	0.3171	0.9711
5	0.2888	0.9888
10	0.2345	0.9905
30	0.0879	0.9912
Scaling		
0.25	0.3073	0.9831
0.5	0.1765	0.9987
0.9	0.0913	0.9990
1.2	0.0188	0.9984
Horizontal translation (128 pixels)	0.0863	0.9971
Vertical translation (128 pixels)	0.0654	0.9970
Gaussian noise		
μ=0,σ=0.001	1.0	1.0
μ=0,σ=0.01	0.9998	0.9998
Salt and pepper noise		
μ=0,σ=0.001	1.0	0.9998
μ=0,σ=0.1	1.0	0.9998
JPEG		
80	1.0	1.0
60	0.9998	0.9998
40	0.9681	0.9681
Low-pass Gaussian filtering		
σ=0.5,7×7	1.0	1.0
σ=0.6,7×7	0.9975	0.9975

**Table 13:** Robustness comparison measured in terms of NC for Lena image.

Attack	[26]	Proposed Method
Rotation (50°)	0.8630	0.9891
Gaussian noise (0,0.001)	0.9971	1.0
Gaussian noise (0,0.005)	0.9792	1.0
JPEG (50)	0.9938	0.9832
Salt and pepper noise (0,0.001)	0.9959	1.0
Salt and pepper noise (0,0.005)	0.9985	1.0
Median filter (3×3)	0.9942	0.9802
Histogram equalization	0.8530	1.0

**Table 14:** Robustness comparison measured with [27] in terms of NC for Lena image.

Attack	[27]	Proposed Method
Gaussian noise (0,0.005)	0.8822	1.0
Gaussian noise (0,0.3)	0.8894	0.9002
Salt and pepper noise (0,0.001)	0.9770	1.0
Rotation (20°)	0.9803	0.9994
Rotation (50°)	0.9719	0.9991
JPEG (40)	0.9776	0.9681
JPEG (30)	0.9701	0.9532
Median filter (3×3)	0.9758	0.9802
Histogram equalization	0.9854	1.0

**Table 15:** Robustness comparison measured with [28] in terms of NC for Lena image.

Attack	[28]	Proposed Method
Gaussian noise (0,0.001)	0.9988	1.0
Gaussian noise (0,0.01)	0.9830	0.9998
Salt and pepper noise (0,0.1)	0.9877	0.9903
Salt and pepper noise (0,0.5)	0.9770	0.9778
Rotation (10°)	0.9858	0.9995
Rotation (20°)	0.9851	0.9994
Rotation (30°)	0.9853	0.9987
Rotation (40°)	0.9872	0.9990
Rotation (50°)	0.9881	0.9991
JPEG (90)	0.9990	1.0
JPEG (60)	0.9990	0.9999
Median filter (3×3)	0.9962	0.9802
Histogram equalization	0.9972	1.0

**Table 16:** Robustness comparison with [13] measured in terms of NC for Lena.

Attack	[13]	Proposed Method
Scaling (0.25)	0.9774	0.9831
Scaling (0.5)	0.9919	0.9987
Scaling (0.9)	0.9931	0.9990
Scaling (1.2)	0.9906	0.9984
Rotation (2°)	0.9741	0.9998
Rotation (5°)	0.9813	0.9998
Rotation (10°)	0.9861	0.9995
Rotation (30°)	0.9861	0.9987
Rotation (45°)	0.9828	0.9903
Horizontal cycling translation (128 pixels)	0.9964	0.9971
Vertical cycling translation (128 pixels)	0.9964	0.9970
JPEG (100)	0.9966	1.0
Median filter (3×3)	0.9913	0.9802
Center cropping (25%)	0.9179	1.0
Gaussian noise (0,0.01)	0.9788	1.0
Gaussian noise (0,0.05)	0.9509	1.0
Salt and pepper noise (0,0.01)	0.9758	1.0
Salt and pepper noise (0,0.05)	0.9644	0.9998

**Table 17:** Comparison of the robustness of the proposed algorithm with different methods against several attacks for Lena image.

Attack	[21]	[13]	Proposed Method
Median filter (3×3)	0.5170	0.9913	0.9802
Cropping 25%	0.9822	0.9179	1.0
JPEG			
100	0.9941	0.9966	1.0
Rotation			
2°	0.9687	0.9741	0.9711
5°	0.9197	0.9813	0.9888
10°	0.7825	0.9861	0.9905
Scaling			
0.9	0.9710	0.9931	0.9990
1.2	0.8709	0.9906	0.9984

## Data Availability

Not applicable.

## Appendix A

**Table A1:** Robustness comparison between Hu’s method [34] and the proposed scheme in terms of NC.

Attack	Hu’s Method [34]	Proposed Method
Cropping (50%)	0.9755	0.9897
Rotation (45°)	0.9861	0.9803
Gaussian noise (0,0.01)	0.9927	1.0
Histogram equalization	0.9817	1.0
JPEG (70)	0.9975	1.0

**Table A2:** Robustness comparison with [29] measured in terms of NC for Lena.

Attack	[29]	Proposed Method
Rotation (2°)	0.9396	0.9998
Rotation (5°)	0.9308	0.9998
Rotation (10°)	0.8861	0.9995
JPEG (100)	0.9818	1.0
Median filter (3×3)	0.6450	0.9802
Center cropping (25%)	0.9743	1.0
Center cropping (50%)	0.9803	1.0
Center cropping (75%)	0.9803	0.9998
Salt and pepper noise (0,0.001)	0.9803	1.0
Salt and pepper noise (0,0.005)	0.9698	1.0
Salt and pepper noise (0,0.01)	0.9494	1.0
Salt and pepper noise (0,0.02)	0.9282	0.9998

**Table A3:** Robustness comparison with [44] measured in terms of NC for Lena.

Attack	[44]	Proposed Method
Salt and pepper (0.001)	0.9902	0.9907
Gaussian (μ=0, σ=0.3)	0.2631	0.8102
Median filtering (3×3)	0.9994	0.9802
Center Cropping (25%)	0.2793	1.0
Histogram equalization	0.1981	1.0
JPEG (Q = 100)	0.9696	1.0
JPEG (Q = 50)	0.9998	0.9999
JPEG (Q = 40)	0.9976	0.9681
JPEG (Q = 30)	1.0	0.9535
Rotation 2°	0.9914	0.9998
Rotation 5°	0.9932	0.9998
Rotation 10°	0.9811	0.9995
Scaling (0.5)	0.8350	0.9987
Scaling (0.9)	0.9996	0.9990
Scaling (1.2)	0.9997	0.9984

**Table A4:** Robustness comparison with [45] measured in terms of BER for Lena, Baboon and Peppers images.

	[45]			Proposed Method		
**Attack**	**Lena**	**Mandrill**	**Peppers**	**Lena**	**Mandrill**	**Peppers**
JPEG (Q = 40)	0	0.0667	0.0833	0	0.0105	0.0154
JPEG (Q = 30)	0	0.0833	0.1167	0	0.0048	0.0012
JPEG (Q = 20)	0	0.0667	0.2833	0.0127	0.0210	0.0267
JPEG (Q = 10)	0.0667	0.2333	0.4167	0.0354	0.0283	0.0391
scaling 0.6	0	0.0500	0.0333	0	0.0249	0
scaling 0.5	0	0.0500	0.0500	0	0.0108	0.0102
scaling 0.4	0	0.0333	0.2167	0	0.0045	0.0068
Cropping 30%	0	0	0	0	0	0
Cropping 40%	0	0	0	0	0	0
Cropping 50%	0	0	0	0	0	0
Rotation 15°	0	0	0	0	0	0
Rotation 30°	0	0	0	0	0	0
Rotation 45°	0	0	0	0	0.0011	0.0009
Median filtering (3×3)	0	0.0667	0	0	0.0027	0

**Table A5:** Robustness comparison with [34] measured in terms of NC for Lena, Peppers, and Baboon images.

Attack	Method					
	[34]			Proposed Method		
	Lena	Peppers	Baboon	Lena	Peppers	Baboon
CR (50%)	0.9755	0.9775	0.9805	1.0	1.0	1.0
ROT(45)	0.9861	0.9814	0.9683	0.9903	0.9804	0.9817
GN (1%)	0.9927	0.9945	0.9885	1.0	1.0	1.0
HE	0.9817	0.9927	0.9499	1.0	1.0	1.0
JPEG (70)	0.9975	0.9977	0.9976	1.0	1.0	1.0
JPEG2000 (70)	0.9976	0.9976	0.9967	0.9999	1.0	0.9999

**Table A6:** Robustness comparison with [24] measured in terms of NC for Lena, Peppers, and Baboon images.

Attack	Method					
	[24]			Proposed Method		
	Lena	Peppers	Baboon	Lena	Peppers	Baboon
CR (50%)	1.0	1.0	0.9999	1.0	1.0	1.0
ROT(45)	0.2874	0.3120	0.1985	0.9903	0.9804	0.9817
GN (1%)	1.0	1.0	1.0	1.0	1.0	1.0
HE	1.0	1.0	1.0	1.0	1.0	1.0
JPEG (70)	0.9998	0.9997	0.9998	1.0	1.0	1.0
JPEG2000 (70)	0.9784	0.9802	0.9778	0.9999	1.0	0.9999

**Table A7:** Average BER values after several attacks applied to thirty test images.

Attacks	BER
Histogram equalization	0
Gaussian noise (μ=0 ,σ=0.001)	0.0001
Gaussian noise (μ=0 ,σ=0.005)	0.0022
Salt & pepper noise (μ=0 ,σ=0.001)	0.0018
Low-pass Gaussian filtering	
(σ=0.5,3×3)	0.0009
(σ=0.5,5×5)	0.0011
(σ=0.5,7×7)	0.0022
(σ=0.5,9×9)	0.0044
(σ=0.6,3×3)	0.0075
(σ=0.6,5×5)	0.0106
(σ=0.6,7×7)	0.0169
(σ=0.6,9×9)	0.0283
Gaussian smoothing	
(σ=0.5,3×3)	0.0014
(σ=0.5,5×5)	0.0058
(σ=0.5,7×7)	0.0157
(σ=0.5,9×9)	0.0237
(σ=0.6,3×3)	0.0281
(σ=0.6,5×5)	0.0328
(σ=0.6,7×7)	0.0382
(σ=0.6,9×9)	0.0402
JPEG compression	
90%	0
80%	0.0012
75%	0.0023
70%	0.0058
60%	0.0087
50%	0.0105
40%	0.0204
30%	0.0210
20%	0.0321
10%	0.0432
JPEG2000 compression	
CR = 2	0
CR = 4	0
CR = 6	0.0023
CR = 8	0.0102
CR = 10	0.0289
Cropping	
25%	0
50%	0.0001
Scaling	
0.5%	0.0003
1.5%	0.0097
Rotation	
10%	0.0001
15%	0.0004
25%	0.0086
40%	0.0104
Combination attacks	
HE + GN (σ=0.001)	0.0092
HE + GN (σ=0.01)	0.0201
HE + SPN (σ=0.001)	0.0102
HE + SPN (σ=0.01)	0.0225
GN (σ=0.001) + JPEG compression (90%)	0.0098
GN (σ=0.001) + JPEG compression (70%)	0.0100
SPN (σ=0.001) + JPEG compression (90%)	0.0140
SPN (σ=0.001) + JPEG compression (70%)	0.0190
LPGF (σ=0.5, window size (9×9)) + SPN (σ=0.001)	0.0228
LPGF (σ=0.6, window size (9×9)) + SPN (σ=0.001)	0.0328

**Table A8:** Robustness after several attacks applied to thirty test images.

Attacks	NC
Histogram equalization	1
Gaussian noise (μ=0 ,σ=0.001)	0.9904
Gaussian noise (μ=0 ,σ=0.005)	0.9889
Gaussian noise (μ=0 ,σ=0.8)	0.7885
Salt & pepper noise (μ=0 ,σ=0.001)	0.9907
Salt & pepper noise (μ=0 ,σ=0.7)	0.7653
Low-pass Gaussian filtering	
(σ=0.5,3×3)	0.9975
(σ=0.5,5×5)	0.9973
(σ=0.5,7×7)	0.9958
(σ=0.5,9×9)	0.9934
(σ=0.6,3×3)	0.9879
(σ=0.6,5×5)	0.9861
(σ=0.6,7×7)	0.9836
(σ=0.6,9×9)	0.9812
Gaussian smoothing	
(σ=0.5,3×3)	0.9982
(σ=0.5,5×5)	0.9971
(σ=0.5,7×7)	0.9958
(σ=0.5,9×9)	0.9934
(σ=0.6,3×3)	0.9891
(σ=0.6,5×5)	0.9888
(σ=0.6,7×7)	0.9883
(σ=0.6,9×9)	0.9802
(σ=10,9×9)	0.2184
JPEG compression	
90%	1
80%	0.9997
70%	0.9918
60%	0.9825
50%	0.9785
40%	0.8981
30%	0.7710
20%	0.7674
10%	0.7355
5%	0.7182
3%	0.4985
JPEG2000 compression	
CR = 2	1.0
CR = 4	0.9913
CR = 6	0.9137
CR = 8	0.8752
CR = 10	0.7031
Cropping	
25%	1.0
50%	0.9962
Scaling	
0.5%	0.9903
1.5%	0.9872
Rotation	
10%	0.9999
15%	0.9994
25%	0.9896
40%	0.9877
Combination attacks	
HE + GN (σ=0.001)	0.9968
HE + GN (σ=0.01)	0.9895
HE + SPN (σ=0.001)	0.9888
HE + SPN (σ=0.01)	0.9862
GN (σ=0.001) + JPEG compression (90%)	0.9998
GN (σ=0.001) + JPEG compression (70%)	0.9891
SPN (σ=0.001) + JPEG compression (90%)	0.9933
SPN (σ=0.001) + JPEG compression (70%)	0.9910
LPGF (σ=0.5, window size (9×9)) + SPN (σ=0.001)	0.9834
LPGF (σ=0.6, window size (9×9)) + SPN (σ=0.001)	0.9801
